# Uncovering the fruit bat bushmeat commodity chain and the true extent of fruit bat hunting in Ghana, West Africa

**DOI:** 10.1016/j.biocon.2011.09.003

**Published:** 2011-12

**Authors:** A.O. Kamins, O. Restif, Y. Ntiamoa-Baidu, R. Suu-Ire, D.T.S. Hayman, A.A. Cunningham, J.L.N. Wood, J.M. Rowcliffe

**Affiliations:** a Cambridge Infectious Disease Consortium, Dept. of Veterinary Medicine, University of Cambridge, Madingley Road, Cambridge CB3 0ES, UK; b Institute of Zoology, Zoological Society of London, Regent’s Park, London NW1 4RY, UK; c Centre for African Wetlands, University of Ghana, P. O. Box LG 67, Legon, Accra, Ghana; d Wildlife Division of the Forestry Commission, Accra, Ghana

**Keywords:** Bushmeat hunting, Commodity chain, Ghana, *Eidolon helvum*, Fruit bat hunting

## Abstract

Harvesting, consumption and trade of bushmeat are important causes of both biodiversity loss and potential zoonotic disease emergence. In order to identify possible ways to mitigate these threats, it is essential to improve our understanding of the mechanisms by which bushmeat gets from the site of capture to the consumer’s table. In this paper we highlight the previously unrecognized scale of hunting of the African straw-colored fruit bat, *Eidolon helvum*, a species which is important in both ecological and public health contexts, and describe the commodity chain in southern Ghana for its trade. Based on interviews with 551 Ghanaians, including bat hunters, vendors and consumers, we estimate that a minimum of 128,000 *E. helvum* bats are sold each year through a commodity chain stretching up to 400 km and involving multiple vendors. Unlike the general bushmeat trade in Ghana, where animals are sold in both specialized bushmeat markets and in restaurants, *E. helvum* is sold primarily in marketplaces; many bats are also kept by hunters for personal consumption. The offtake estimated in this paper raises serious conservation concerns, while the commodity chain identified in this study may offer possible points for management intervention. The separation of the *E. helvum* commodity chain from that of other bushmeat highlights the need for species-specific research in this area, particularly for bats, whose status as bushmeat is largely unknown.

## Highlights

► We study how fruit bats are hunted and sold as bushmeat in Ghana, West Africa. ► Globally, bats are under-represented in market reviews, and threatened by hunting. ► 128,000 *Eidolon helvum* are sold each year in southern Ghana. ► Fruit bats do not follow the normal commodity chain for bushmeat. ► *E. helvum* may be missed by market surveys and threatened by this level of hunting.

## 1 Introduction

The consumption of wild animals as meat, a product often called bushmeat, poses challenges for both wildlife conservation and human well-being. These challenges include depletion of threatened and endangered species (Milner-Gulland et al., 2003); the transmission of zoonotic diseases (Daszak et al., 2000; Wolfe et al., 2005); threats to the food and economic security of some of the poorest countries (Bennett et al., 2007); and the loss of vital ecosystem services, like pollination and reforestation, that are necessary for human wellbeing (Ehrlich and Wilson, 1991; McConkey and Drake, 2006).

As people frequently are dependent on bushmeat as a vital source of protein or income (Wilkie et al., 2011), it is necessary to assess the sustainability of bushmeat hunting before population crashes damage both human and ecosystem health. Using bushmeat marketplaces to monitor the status of hunted species (see Rowcliffe et al., 2003, Juste et al., 1995, Fa et al., 2003) circumvents many of the resource limitations of other, more direct techniques; however, it is also dependent on all species of concern making it to market. A growing body of research is beginning to answer questions of the species involved, extraction rates, impacts on biodiversity and socioeconomic aspects of the bushmeat trade (for example see Mbete et al., 2011; Wilcox and Nambu, 2007); but the structure of this trade is still poorly documented. Unraveling the commodity chain from capture to consumption for each species involved is vital to understanding how best to manage the trade, mitigate its potentially deleterious effects and powerfully utilize techniques like market surveys (Cowlishaw et al., 2005b).

Here we consider the bushmeat trade of the African straw-colored fruit bat, (*Eidolon helvum*) in Ghana. Little is known of the use of this species as bushmeat, and 31 supposedly comprehensive bushmeat survey papers failed to report anything on bats (Mickleburgh et al., 2009). A number of reports, however, mention the massive threat many species of fruit bats face as a result of severe overhunting (e.g. Struebig et al., 2007), and the IUCN Red List reports *E. helvum* as a near-threatened species due to overhunting (IUCN, 2010). The disparity between the lack of fruit bats being recorded in markets in Ghana where other types of bushmeat are found for sale (e.g. Cowlishaw et al., 2005b) and the reports of overhunting may indicate that bats do not follow a typical bushmeat commodity chain and standard bushmeat surveys may therefore be underestimating impacts. *E. helvum*, like many bat species, is especially vulnerable to hunting, due to a slow reproductive rate (Mutere, 1965). It is also an extremely widespread and highly mobile species (DeFrees and Wilson, 1988; Richter and Cumming, 2005) which could, in principle, help to reduce impacts if hunting is localized and limited. However, *E. helvum* congregates in large, predictable roosts (Happold and Happold, 1978), exposing a large proportion of the wider population to hunting, and likely resulting in widespread impacts across the range.

Furthermore, *E. helvum* plays an important role in seed dispersal and regeneration of valuable natural products (Taylor et al., 2000; Kankam and Oduro, 2009); overhunting of *E. helvum* could severely damage its ability to provide these vital ecosystem services. Another significant concern is that *E. helvum* may be host to several zoonotic or potentially-zoonotic infections of high public health importance, including henipaviruses (Hayman et al., 2008a), lyssaviruses (Kuzmin et al., 2008; Hayman et al., 2008b) and Ebola virus (Hayman et al., 2010). Unveiling the extent of the commodity chain would be the first step towards the identification of potential risk groups for zoonotic transmission.

We undertook this study at a number of sites across southern Ghana. The initial focus for the study was the city of Accra, which holds a massive seasonal colony of 250,000–1 million *E. helvum* individuals (Hayman, 2008), and where we had observed local hunting and consumption of *E. helvum* prior to this study. Given the large numbers and dense, highly visible roosting of *E. helvum* in Accra in comparison to other bat species, we expected that it would be the predominant bat species used as bushmeat in the city. There is a notable scarcity of bats in published reports of the bushmeat trade in Ghana (e.g. Brashares et al., 2004; Cowlishaw et al., 2005b), which led us to hypothesize that the use of bats as bushmeat had been underestimated. The primary aim of our study, therefore, was to evaluate the numbers of fruit bats in general, and *E. helvum* in particular, that are hunted or sold in southern Ghana as bushmeat. If our hypothesis of a previous underestimation of the presence of fruit bats in the bushmeat trade was confirmed, we aimed to understand why this might have occurred. Finally, we sought to establish if hunted *E. helvum* passed through the same commodity chain as other Ghanaian bushmeat, and from where these bats were sourced.

## 2 Materials and methods

### 2.1 Study area

We examined two cities: Accra (5°33′N, 0°11′W) and Kumasi (6°41′N, 1°37′W); a town: Nkawkaw and surrounding villages (6°33′N, 0°46′W) and two broader localities in the Volta Region (7°12′N, 0°19′W), and the Afram Plains (7°2′N, 0°4′W), all located within southern Ghana. Accra is the capital of Ghana, with an urban population of 1.6 million in the 2000 census (Ghana Statistical Service, 2002). With 1.4 million inhabitants, Kumasi is the second largest city in Ghana, located to the northwest of Accra in the Ashanti region (Ghana Statistical Service, 2002). In Kumasi there is a large central market, where bushmeat is sold, as well as a bushmeat-specific market. Nkawkaw is a small, rural town in the hills of the Eastern Region, located along a major road that runs from Accra to Kumasi. Markets in this region are very small, usually supplying a single village. The Volta Region stretches along the eastern border of southern Ghana. Each town in the Volta Region has a central market that is active on a given market day each week. The Afram Plains lie in the easternmost part of the Eastern region, nestled into the western shores of Lake Volta. As in other parts of Africa (Milner-Gulland et al., 2003), farmers in the Volta Region and the Afram Plains often hunt bushmeat for food, for additional income and to protect their crops.

### 2.2 Questionnaires

In order to obtain information about the use of bats as bushmeat, we conducted face-to-face interviews using a standardized questionnaire. Since previous studies had not shown extensive bat hunting in Ghana, we initially directed interviews to hunters we witnessed shooting *E. helvum* and to vendors selling *E. helvum* in the markets. These interviewees provided details of other hunters and vendors, and thus we were able to penetrate the entire commodity chain through a cascade effect. In addition, we conducted convenience sampling in each of our sites by standing at the entrance to each main market place, or along the only main road of each small village, in our study sites and by choosing for interview the first person who walked by at exactly 5 min after we had completed our previous interview. All interviews were conducted in person first in English, with a local Ghanaian translator translating if the interviewee did not understand something. Fruit bats are legal to hunt, and there is no taboo or stigma associated with consuming bats across southern Ghana. All interviewees were relaxed and comfortable answering our questions.

Interviews were conducted in November 2009 and February 2010, and these enabled us to trace the bat bushmeat commodity chain in southern Ghana. From our interviews, we identified four actor types involved in the bat bushmeat trade: hunters, vendors, chopbar (local restaurant) owners and individual consumers. We further identified hunters as either (1) subsistence hunters, who hunted bats only for personal or family consumption, or (2) commercial hunters, who hunted bats to obtain an income. We defined vendors as individuals who retailed bats that someone else had captured. We classified primary vendors as those who bought bats from hunters, and secondary vendors as those who bought bats only from other vendors. Vendors were predominantly located in markets, although a few were “wandering vendors” in the street. We also distinguished between “active” and “inactive” hunters and vendors, using activity within the last 12 months as the cut-off between the two states.

Our questionnaire design was informed by a previous survey of bushmeat trade in southwestern Ghana (Cowlishaw et al., 2005b). We used a standard questionnaire, comprised of both multiple-choice and open-ended questions, for all respondents. This questionnaire enquired about demographic information of the respondent, interactions with bats (e.g. hunting or eating of bats), beliefs about bat bushmeat, perceptions of disease risks from bats, and general meat preferences. We asked any respondent that sold or hunted bats to complete additional questionnaires relating to these activities, which asked for details such as the frequency and locations of bat hunting or purchasing of bats. We interviewed a total of 551 people in southern Ghana (Table 1). We also visited a variety of market places in an attempt to assess the numbers and species of any bats being sold.

### 2.3 Analyses

The sporadic occurrence of fruit bats in bushmeat markets ultimately made direct market counts unfeasible as a means of estimating numbers of bats traded. We therefore used interview responses to estimate the numbers of bats hunted (*N_H_*) and sold (*N_S_*) annually for each respondent, using the product of the number hunted or sold per day active (*H* or *S* respectively), the frequency of hunting or selling (days per month, *D_H_* or *D_S_* respectively), and the length of the hunting or selling season (months, *L*):

$$N_{H}={\mathrm{HD}}_{H}L$$

$$N_{S}={\mathrm{SD}}_{S}L$$

If a range was given for a single answer, we used the mid-point value.

For the numbers per day active, we used the response to the question “How many bats do you catch/sell on a normal day?” (*H* or *S*), which yielded the most responses. We also asked “How many bats did you catch/sell yesterday or the last time you hunted/sold bats?” (*H_L_* or *S_L_*) and “how many did you catch/sell today?” (*H_T_*) but many of the respondents were unable to recall the answer for the “last time” question. We did two linear regressions to compare hunter responses to the different questions, which demonstrated limited bias between the sets of answers (*H_L_* = 0.99*H* − 0.28, *R*^2^ = 0.99, *n *= 33; *H_T_* = 1.20*H* − 6.50, *R*^2^ = 0.60, *n *= 9).

Only three vendors had sold anything on the day of the interview, so this question was not considered for analysis. Linear regression did show a reporting bias between the answers for the “normal” day number of bats sold and how many bats the vendor sold on the last day they had sold bats (*S_L_* = 0.15*S* + 64.26, *R*^2^ = 0.56, *n *= 13). If they were unsure of the length of the hunting/selling season, or gave a wide range, we used the shortest consistent season length reported (3 months). Summing across respondents gave us minimum numbers of bats hunted and sold in the region annually; we cannot estimate the true totals because we were unable to estimate the total numbers of actors involved due to the dispersed nature of fruit bat hunting and selling across southern Ghana.

We also created a simple model to estimate the current population size that would be needed to support current offtake rates for a given intensity of exploitation at equilibrium. Using a logistic population model, the yield at equilibrium is:

$$Y={\mathrm{Nr}}_{\max}(1-N/K)$$

(Milner-Gulland and Rowcliffe, 2007) where *N* is population size, *K* is the population carrying capacity, and *r*_max_ is the intrinsic rate of increase. We use the ratio *N*/*K* as an indicator of the impact of exploitation, with a value of 0.5 indicating a population exploited to maximum sustainable yield (MSY), values close to 0 indicating overexploitation and values close to 1 indicating light exploitation. In this way, we avoid the difficulty of having to estimate *K* by making it implicit in the definition of degree of exploitation. We explore three exploitation impact scenarios: high impact (*N*/*K *= 0.2), MSY (*N*/*K *= 0.5) and low impact (*N*/*K *= 0.8). Solving for *N* gives us:

$$N=\frac{Y}{r_{\max}(1-N/K)}$$

which allows us to estimate how large the current population would have to be at equilibrium to support a range of potential offtake rates given *r*_max_ values. While the necessary life history parameters are not yet precisely defined for *E. helvum*, we calculated a range of plausible *r*_max_ values based on data from Hayman (2008 and unpublished) and biological intuition, and using a 3-stage post-breeding matrix model. Assuming that, under ideal conditions, all females produce one young per year from their second year on, productivity for the second and third stages is 0.5. Adult survivorship without hunting is likely in the region of 0.8–0.9, while first year survivorship is lower, probably between 0.6 and 0.8. This range yields *r*_max_ values between about 0.05 and 0.15.

**Fig. 1:**
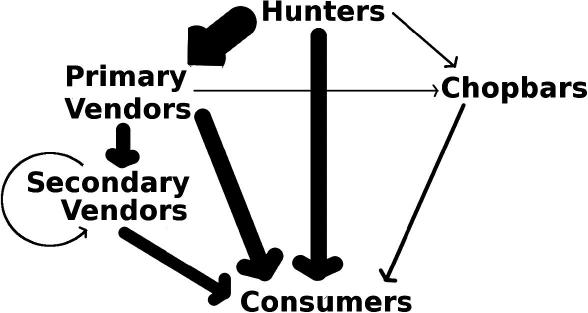
The basic structure of the bat bushmeat commodity chain in southern Ghana. The width of arrows is proportional to the relative proportions of reported connections between each pair of actors along the chain*.*

In order to approximate the relative volumes of flow of bat bushmeat along different trade routes, we used the reported relationships among the actors. We constructed a weighted network, where the weight of a connection pointing towards a customer type was proportional to the number of respondents stating that they sold bats to that particular customer type (see Fig. 1 in results). As we had little information on the numbers of bats sold to the different categories, each respondent was weighted equally; when respondents sold to multiple actors, their weighting was divided evenly among actors. For example, a hunter who sold to both consumers and vendors was counted as 0.5 for each. Since all bats had to come from hunters, we scaled the total weight of connections from hunters to 100%. This was then allocated according to the number of hunters reporting selling to each type of consumer: 64% to primary vendors, 32% to consumers and 4% to chopbars. The 64% given to primary vendors was then further distributed proportionally to the proportion of reported customer types for primary vendors. This process was carried out for all reported pathways to consumers.

## 3 Results

In Ghana, fruit bats are sold dead, and often smoked. We located fewer than 100 bat carcasses being sold in marketplaces, and only observed two hunters with fresh kills. However, not only did we identify the bats in every case as *E. helvum*, but all hunters and vendors who knew the Accra colony identified the bats being hunted or sold as the same species. Additionally, many hunters and vendors pointed out that insectivorous bats smelled and tasted unappetizing, and are thus never eaten. Therefore, we use “bat bushmeat” to refer almost exclusively to *E. helvum* as this species seems to form the vast majority of all bats hunted and sold in southern Ghana.

### 3.1 Extent of fruit bat hunting and bushmeat trade

We estimated that the 37 active vendors interviewed in the study area sold more than 128,000 bats per year (Table 2). The 46 active hunters we interviewed reported hunting approximately 44,000 bats per year (Table 3). It is possible that a limited number of bats were counted twice: once by primary vendors, and again by secondary vendors. Accra vendors who bought only from the Volta region, and thus whose bats could have been potentially double-counted, accounted for 8312 of the estimated sold bats. Two other vendors, who accounted for another approximately 8000 bats, bought bats from the Volta region as well as from elsewhere, but were unable to account for how many bats came from each source.

In the Volta region, some of the hunters questioned traveled with other hunters (whom we did not interview) to a single island called Biobio in Lake Volta. Each responding hunter who hunted there was able to give the total number of bats hunted by his whole group, as the hunters usually shot the bats as a team, counted up the bats killed and divided them equally among the participants. Only one member per hunting group was interviewed, although there were many such groups from which no hunter was interviewed at all. By totaling the reported numbers of bats hunted by each group, we estimated that the six groups represented in our interviews harvested at least 40,000 bats from Biobio each year.

From our convenience sampling of men, the highest proportions reporting that they had hunted bats were in the Afram Plains (38% of respondents) and the Volta region (29%), with Accra third (25%) and Kumasi and Nkawkaw equally with the lowest proportion (21% each).

Out of 95 hunters and 48 vendors questioned, 85% and 96% respectively reported bats as seasonal meat, with November to March most commonly listed by both groups as the in-season period. There were two dominant reasons given for this ‘seasonality.’ The first explanation was that the *E. helvum* seem to migrate seasonally, and are not present to be hunted. The second reason given was that hunters were unable to hunt them due to limited time from their main occupation, such as occurred during the farm harvest season.

**Fig. 2:**
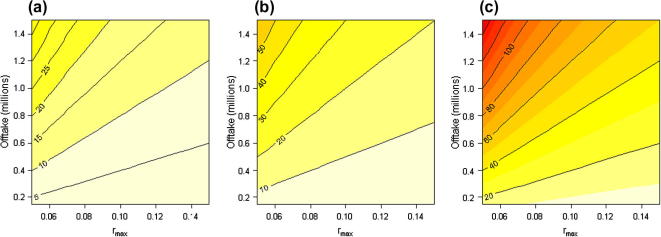
Estimated equilibrium *E. helvum* population sizes necessary to sustain: (a) heavy impact (*N*/*K *= 0.2), (b) maximum sustainable yield (*N*/*K *= 0.5), and (c) light impact (*N*/*K *= 0.8), in relation to intrinsic rate of increase (*r*_max_) and annual numbers removed by hunting (offtake). Contour lines give equilibrium population, *N*, in millions, and scales differ between graphs, while shading is in 10 million intervals, consistent across graphs.

We visited only 9 of 163 towns listed on Google Maps for the Volta region, and therefore expect that our minimum estimate of current harvest of 128,000 bats per year is likely to be a substantial underestimate, perhaps by as much as an order magnitude. We therefore explored offtake rates between 128,000 and 1.5 million in our model of population sizes and exploitation pressure (Fig. 2). In these explorations, the light impact scenario assumes that the current, equilibrium population is close to carrying capacity (*N*/*K* = 0.8), so it is higher than in the MSY or high impact scenarios. Assuming mid-range estimates of offtake (0.8 million) and *r*_max_ (0.1), current populations needed to support this level of offtake assuming heavy impact, MSY and light impact would be around, respectively, 10, 17 or 40 million. Scenarios, assuming annual offtake and *r*_max_ values at the extremes of the ranges explored suggest equilibrium populations of between about 10 and 120 million for light impact (*N*/*K *= 0.8), or between about 2 and 30 million for heavy impact (*N*/*K *= 0.2).

### 3.2 Commodity chain structure and actors involved

Part-time commercial bat hunters and subsistence gatherers hunted fruit bats (Table 4). Commercial hunters sold their bats to market vendors. All hunters were men (*n *= 95) and all but one of the vendors (*n *= 48) were women. Some primary vendors sold bats to secondary vendors. In the Volta region, 88% of hunters were active (had hunted bats in the previous 12 months) while 74% of hunters were active in Afram, 50% in Accra, 40% in Nkawkaw and 33% in Kumasi.

All vendors interviewed in Accra and Kumasi were marketplace vendors with an established booth at a fixed location, and who only sold at their local marketplace. In Nkawkaw, three of the seven active vendors moved from town to town, or sent children out to sell bats in different places. In the Volta region, three vendors reported traveling down to Accra to sell their bats personally in the Accra markets.

The majority (60%, Fig. 1) of commercial hunters interviewed sold bats to vendors, while the remaining 40% sold directly to consumers or chopbars. Forty-three percent of primary vendors interviewed sold to secondary vendors and 50% sold to consumers. Almost 80% of secondary vendors sold to consumers. Only in Accra did any vendors report selling to chopbars, and we located chopbars that sold bat bushmeat only in Accra. In Accra, three out of the five active, commercial hunters reported selling to particular customers who sought them out deliberately and pre-arranged bat bushmeat orders. In the Volta region, three vendors supplied particular hunters with shotgun cartridges to secure a supply of bats. Of the 10 active, commercial hunters interviewed in Afram Plains, three of these hunters sold bats to Accra vendors. Out of the 21 active, commercial hunters interviewed in the Volta region, one fifth sold bats directly to vendors from Accra. Additionally, one third of the Volta hunters would sell to the same vendor to whom they had sold previously rather than to any vendor they could find.

**Fig. 3:**
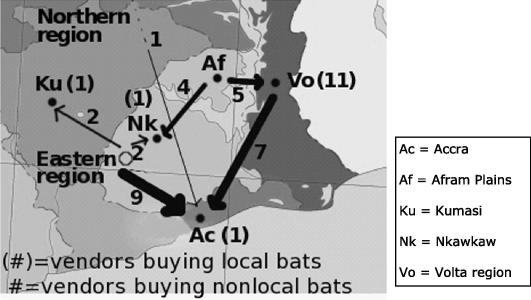
The origins of bat bushmeat bought by vendors. Arrows originate at the reported source of bats and are to scale for the number of vendors interviewed in the receiving site. The numbers adjacent to arrows indicate the number of vendors in the receiving locale purchasing bats from the corresponding source location, while the numbers in parentheses indicate vendors in each site who purchased local bats.

The commodity chain through which fruit bats are sold extends beyond Accra and local hunters (Fig. 3). Information obtained from market vendors in Accra about buying fruit bats from the Volta and Eastern regions was corroborated by vendors in these regions who reported selling bats to visiting vendors from Accra or traveling to Accra to sell to vendors there. When they did not know the exact origin of the bats they sold, vendors reported them as coming from either the “Eastern region” or the “Northern region.” The single vendor reporting buying from the Northern region was the only mention of bats from outside of the study area.

**Fig. 4:**
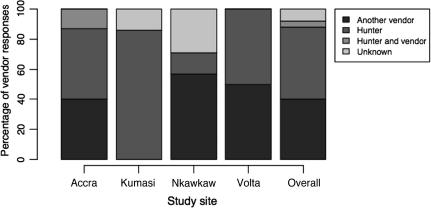
Breakdown, as a percentage, of each type of supplier to vendors in each study site.

The pattern of vendor suppliers varied throughout the study area (Fig. 4). Vendors who sold to other vendors paid a marginally lower amount of money to procure bats and sold marginally more bats than vendors who sold only to individual consumers. In Accra, the mean number of bats sold annually per vendor who only sold to other vendors was 3200, while the mean number of bats sold annually per vendor selling only to the public was 2800. In the Volta region, “vendor-only” sellers sold a mean of 2600 bats annually, whilst vendors selling to both the public and to other vendors sold 2500, and vendors selling only to the public sold a mean of 1900 bats annually. There were, however, no other observable differences amongst the different types of vendors and the vendors themselves reported that there were no identified wholesalers and no discounts for buying bats in large quantities. Vendors reported buying as many bats as their available cash flow would allow at any given time and all but one vendor reported having a greater customer demand than they could supply.

### 3.3 Income from bat bushmeat

**Fig. 5:**
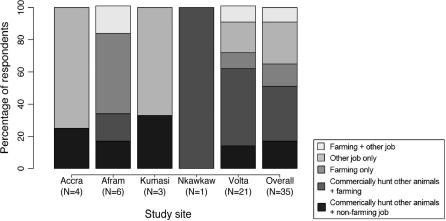
Proportions of types of additional jobs held by commercial bat hunters in each study site.

Out of 39 vendors who responded to the questions on income, 77% said that bats formed “very little” of their income; 18% chose “half” or “some,” and 5% chose “most.” The mean price per kilo for smoked bats (based on 53 bat carcasses) in Accra markets was USD 5.66, compared to a mean price of USD 15.53 per kilo of smoked grasscutter (*Thryonomys swinderianus, n *= 16). Kumasi had the highest buying and selling price as well as highest profit for vendors of all the study sites (Table 5). No vendor sold only bats, and no bat hunter was a full-time bat hunter, or even a commercial bushmeat hunter (Fig. 5).

## 4 Discussion

### 4.1 Numbers of E. helvum harvested

The numbers of *E. helvum* we estimate being harvested not only exceeds previous bushmeat reports, but is likely a substantial underestimate; our survey only covered a fraction of the rural areas where most bat hunting occurs (e.g. we visited only 9 out of 163 towns listed on Google Maps for the Volta region). Therefore the national volumes of offtake and sale might exceed our local estimates by an order of magnitude. Given that the relative risk of extinction among pterododidae species, such as *E. helvum*, is more than 13 times that of other bat clades (Jones et al., 2003), such hunting should be taken very seriously. In Asia, *Pteropus* fruit bats are experiencing serious population declines due to unsustainable hunting (Epstein et al., 2009; Harrison et al., 2011). Indeed, overhunting threatens fruit bats across the world, from the South Pacific (e.g. Craig et al., 1994; Brooke and Tschapka, 2002) to Africa (e.g. Jenkins and Racey, 2008; Funmilayo, 1978), and hunting is listed as the primary cause for concern resulting in IUCN near-threatened status for *E. helvum*. The previously largely unidentified *E. helvum* hunting offtake in Ghana, and a lack of suitable data from which sustainability can be assessed, call serious attention to the need for better surveillance and the very real potential for conservation threats to an already near-threatened species.

Our sustainability model represents a first attempt to understand current hunting impacts, albeit with large margins of uncertainty in key parameters. The likely sustainability of current harvest hinges on the size of the population from which offtake is being drawn, and we have only an approximate idea of minimum *E. helvum* population size in Ghana at present. Further, fruit bats little respect national borders, and populations affected may include fruit bats from a number of neighboring countries. We know of only five significant colonies in Ghana, each holding around half a million bats on average during the dry season, suggesting a minimum of 2.5 million in the population. At this level, it is very plausible that the population is hunted far beyond maximum sustainable yield, given that the overexploited scenario suggested a population of around 10 million would most likely be needed to support current offtake. On the other hand, for the population to be minimally impacted by current hunting, a current population of around 10 is the most optimistic scenario, with something in the region of 40 million more likely. To satisfy this expecation, there need to be either at least an order of magnitude more large colonies than we are currently aware of, or many millions of bats dispersed in smaller groups across the country. Based on current knowledge, both of these scenarios appear highly unlikely, and the population is more likely being overexploited. However, there remains enormous uncertainty, highlighting an urgent need for more precise estimates of current population size, hunting offtake and *E. helvum* demography. We also note that our definition of a Ghanaian population is artificial, since *E. helvum* is extremely widespread and mobile (Richter and Cumming, 2005). Large scale population structure and patterns of movement between sub-populations will also need to be better understood for a full assessment of hunting impacts in this species.

### 4.2 Under-representation of fruit bats in bushmeat surveys

Our estimates of numbers of bats harvested and sold in the bushmeat market supported our hypothesis that fruit bat trade have been severely under-reported in previous studies. Several reasons may explain this finding. First, the commodity chain examined in this study does not seem to be as public as the general market. Previous studies in Ghana recorded little or no informal trade in bushmeat (Ntiamoa-Baidu, 1998); yet in our study three of 15 active Accra hunters reported the majority of their sales were to specific clients that personally requested bats – sales that occurred completely outside the formal market forum. Five vendors in Volta reported specifically buying shotgun cartridges for hunters to supply bats, and then immediately selling these bats to pre-arranged vendors from Accra; a review of Volta marketplaces would completely miss these thousands of bats. There are many potential reasons for this difference in trade, and further research is needed to provide clear explanations. However, our observations offered a few possibilities. The first is that bats are viewed as an opportunistic product, one that can only be secured if the customer takes extra pains to seek it out. Such an opinion would encourage consumers or vendors to directly seek out bat hunters, rather than wait and hope the bat hunters supply bats to the wholesale bushmeat market. Additionally, as no bats passed through a wholesaler, it is conceivable that wholesalers actively refuse to deal with bats, forcing bats to be sold via different avenues. There is evidence that wholesalers prefer to concentrate on larger, more valuable animals (Mendelson et al., 2003).

Another reason why bat trade is likely to be seriously underestimated in other bushmeat surveys is simply that they sell very quickly. Sixteen vendors remarked that the number of bats the hunters or vendors supplied was the limiting factor in how many they could sell; many sold out very soon after receiving shipments of bats. A market surveyor would have to be in the market just when bats were delivered to observe them. Furthermore, bats are often delivered packed into baskets; while each basket contains 200–400 bats, it would be still be quite easy to miss a single basket in the hustle of the marketplace. Even our bat-focused survey only directly observed little over a hundred bats in the market place, while vendors consistently reported selling and hunters reported supplying thousands.

A third reason could be the seasonal and geographic variability of bat bushmeat availability. Cowlishaw et al.’s (2005b) study was conducted from 1 January until 29 February, missing both of the reported peaks in bat availability in southern Ghana. However, January is still in-season for bat bushmeat, so the timing does not fully explain why so few bats were reported in previous studies as compared to the numbers we estimated. It is also possible that fruit-bat seasons may vary across Ghana, as they are largely determined by the migration, foraging and seasonal movement patterns of *E. helvum*.

Finally, an hypothesis that we cannot discard is that of a recent increase in both the hunting and selling of bat bushmeat. Both published (e.g. Cowlishaw et al., 2005a) and personal observations indicate a compositional change in the animal species in Ghanaian market places as larger species are hunted out. Previously, only large animals were brought to market and smaller animals like rats—and potentially bats—were brought straight home (Y.N., personal observations). Thus the large number of bats we recorded in trade could actually result from an increase in the use of small animals as commercial goods. This shift, if confirmed, would be more of a concern for a slow reproducer like *E. helvum* (Fayenuwo and Halstead, 1974) than for other small, more rapidly breeding species like *T. swinderianus*. More directed monitoring of the bat trade will be needed in the future to assess whether, and in what way, patterns of trade are changing.

### 4.3 Bat bushmeat commodity chain

We found that the high numbers of fruit bats sold in southern Ghana traverse a complex and extensive commodity chain that stretches over 12,000 sq. km. *E. helvum* is hunted most commonly in the Volta and Eastern regions, and is either sold locally or shipped to Accra and other areas of bat consumption. While this spread may be less common in more remote areas, it is still remarkable here as the Volta Region is a half-day’s drive from Accra with only a single road accessing the entire region. As reported by Cowlishaw et al. (2005b), for the Takoradi bushmeat market in southwestern Ghana, the main actors in this study were farmer–hunters, market vendors and individual consumers. Unlike most types of bushmeat in the Takoradi bushmeat market, however, bats rarely appeared in chopbars and never as wholesale products. Although bat bushmeat seemed to differ in these respects from other types of bushmeat, our findings closely matched the limited reports of specific bat sales in Takoradi, where bats were hunted only by farmer–hunters and were sold directly from hunters to market vendors rather than through wholesalers (G. Cowlishaw, personal communication).

Food wholesalers in Ghana usually sell items in set bulk amounts or, less commonly, sell products for a discount (Clark, 1994); such wholesalers did not appear at all in this bat bushmeat commodity chain. Vendors reported that no wholesalers existed for bat bushmeat, there was no bulk amount that they had to buy from other vendors, and there was no discount for purchasing more bats. The absence of bat bushmeat wholesalers is particularly interesting as these actors are a key point in the commodity chains reported for other bushmeat (Cowlishaw et al., 2005b) and many other products (Clark, 1994). One reason for the absence of wholesalers may be the small size of bats; in the Takoradi market, wholesalers refused to trade in small vertebrates and invertebrates with the justification that these creatures possess a low value-to-weight ratio; wholesalers prefer to use the limited storage space for larger creatures that, per kilo, can fetch more money (Mendelson et al., 2003). Small size does not preclude regular market vendors from trading in bats, however, because individual customers usually buy small amounts. Bypassing wholesalers has important implications for species surveys, as bats will not be picked up in any wholesaler review, and for managing sale of bushmeat. Wholesalers represent a high volume-to-actor ratio and thus a concentrated and potentially more efficient target for management intervention. However, bat bushmeat in Ghana did seem to be concentrated in areas, if not in wholesalers; the Volta region in particular stands out as an area of high bat hunting and consumption. The highest concentration of vendors supplying other vendors occurred in the Volta region, which likely reflects the region’s importance as a source of bats.

### 4.4 Economics of bat bushmeat

In all of our study areas except the Volta region, the majority of hunters interviewed harvested bats for personal consumption; yet, the substantial numbers of bats sold does not support the idea that they are non-commercial items. This apparent contradiction might be explained by the fact that a significant proportion of bats sent to markets throughout the study area are supplied from the Volta region; subsistence hunters can still dominate in places with large market sales of bats, as the bat bushmeat is shipped in rather than hunted locally.

Profits were fairly similar across the study sites, although they were higher in Kumasi. While the low number of responding vendors in Kumasi may have contributed to this difference, it could also represent an area of relatively high demand with low supply. Interestingly, the reported sale price for hunters in the Volta region closely matched the purchase price for vendors in Accra. This similarity could reflect the large number of Accra vendors purchasing bats from the Volta region.

Although bat bushmeat is a highly seasonal product, rarely providing consistent and sufficient profit for a year-round venture, we found that the profit margin offered a significant source of additional income to bat hunters and vendors. The bias between previous sales and normal sales in the vendors’ reporting could be an additional sign of high variability, and suggests an ultimate limit on the extent to which a vendor can depend on bat bushmeat economically. We were unable to consider in our profit calculations the unknown costs of such factors as market stall space rental, shotgun cartridges, long-distance transport of bats or travel to other markets; however, we estimated that the governmental minimum daily wage of 2.22USD (National Tripartite Committee, 2010) could be achieved by selling fewer than 30 bats a day, even if they only received one-third of the calculated profit per bat. One carpenter pointed out he can make more money in a day of hunting bats than in a week of carpentry. Other studies have found that a bushmeat hunter in Ghana can make 3.5 times the governmental minimum wage and as much as a graduate entering the Wildlife Service (Brown and Williams, 2003; Ntiamoa-Baidu, 1998). Furthermore, the willingness of vendors to buy as many bats as they could find or afford as well as the high retail price suggests that the demand for fruit bats is consistently high. The expected profit and the security of a second source of income from bat bushmeat likely drive the continued use of bats despite their seasonality.

### 4.5 Importance of bat bushmeat

While our data suggest a limited overall dependence on bat bushmeat, *E. helvum* may play an important supportive role in the diets and finances of those who utilize it. Firstly, the vast majority of commercial hunters kept some of their catch for personal consumption, underscoring the role that bats play as an additional, if seasonal, meat source. Even if these bats are not sold because, for example, they are of poor quality, such unsalable products still provide an at-cost protein source. The peak season reported for hunting bats corresponds with the main dry season in Ghana, when agriculture production drops. Dei (1989) found that bushmeat significantly contributed to nutrition and income in rural south-eastern Ghana, especially during the lean agricultural season; even if people do not depend on bats overall, they may be vital at key points of scarcity in the season. Using bushmeat to replace depleted or unavailable food sources is well documented: previous studies have found that demand for bushmeat in general increases in Ghana when fish production drops (Brashares et al., 2004). Even if bushmeat is not a primary food source, it appears to play an important contingency role for meeting nutritional requirements, and fruit bats may be part of this fallback.

If bat bushmeat serves as an important food or income source to Ghanaians, then managing the sustainability of this previously-overlooked harvesting of *E. helvum* takes on new complexities. A program that attempts to limit harvesting of *E. helvum* would have to consider alternative sources of both protein and income, which might not be as readily available as bat bushmeat. Harvested bats as well as hunters and vendors can travel hundreds of kilometers from hunting sites to the ultimate market place. Such an extended market chain means that any effort taken to limit bat hunting in the Afram Plains, for example, would have to consider the market demands of Kumasi or Nkawkaw. While the current study showed a closed network of traffic within southern Ghana, further studies on the extent of bat bushmeat trade are required to elucidate whether similarly extensive commodity chains exist for bat bushmeat elsewhere in Ghana, or elsewhere within the range of *E. helvum*.

## 5 Conclusions

Bushmeat harvesting and its accompanying challenges are not disappearing; rather they are intensifying and threatening numerous animal species as well as the people who depend on such natural resources (Milner-Gulland et al., 2003; Wilkie et al., 2011). Fruit bats in Ghana, and indeed in many places around the world, have been little recognized for the ecosystem services they provide as well as the dangers they face. This study sheds light on previously-unreported levels of hunting that could seriously threaten the long-term viability of *E. helvum* in Ghana. Furthermore, clearly far more people are coming into contact with these fruit bats, and the pathogens that they carry, than previously suspected; the widespread, but under-reported, hunting that occurs in Ghana will pose a serious challenge if any public health measures become necessary. More complete information about the extent of hunting, locations of hunted roosts and the longitudinal impacts on *E. helvum* populations will enable us to inform effective management policies to both benefit *E. helvum* conservation and to contribute to the economic and food security of the growing Ghanaian population.

## Figures and Tables

**Table 1:** Total number of respondents in each study site. Numbers in parentheses indicate number of the total that were actively participating in indicated activity, e.g. 15 (12) means of 15 vendors, 12 had sold bats within the previous 12 months. Respondents can occupy multiple categories.

	Respondent category				Sampling method		Total interviews
	Hunters	Vendors	Consumers	None	Convenience	Cascade	
Accra	32 (15)	15 (12)	55 (42)	141	196	18	214
Afram	23 (16)	0 (0)	38 (17)	26	60	6	66
Kumasi	9 (3)	7 (4)	27 (16)	62	77	15	92
Nkawkaw	5 (2)	8 (7)	31 (18)	42	72	9	81
Volta	26 (22)	18 (14)	90 (42)	16	66	32	98
Total	95 (59)	48 (37)	241 (135)	287	471	80	551

**Table 2:** Estimated characteristics of bat sales by the interviewed vendors in the five study sites.

Study site	Mean number of selling days per month	Mean number of bats sold per vendor per selling day	Mean number of months selling per year	Total number of bats sold per year
Accra	16.8	72.6	4.3	38,452
Kumasi	6.25	26.3	5.5	5920
Nkawkaw	10.4	320	3	55,555
Volta	4.2	510	2	28,480
Overall	8.9	280	3.6	128,407

**Table 3:** Estimated characteristics of bat hunting by the interviewed hunters in the five study sites.

Study site	Mean number of hunting days per year	Mean number of bats hunted per hunting day	Mean number of months per year	Total number of bats hunted
Accra	8.9	10.2	3.9	5070
Afram	10.9	47.0	4.5	15,303
Kumasi	7.0	15.0	5.2	5463
Volta	4.2	292.9	2.9	18,403
Overall	7.6	118.7	3.9	44,239

**Table 4:** Breakdown of all hunters (both active and inactive) by commercial status. Four hunters did not respond to this question.

Reason for hunting bats	Study site (*N*)					
	Accra (29) (%)	Afram (23) (%)	Kumasi (9) (%)	Nkawkaw (4) (%)	Volta (27) (%)	Overall (92) (%)
Only sell	3	17	22	0	11	11
Only personal/family consumption	72	52	56	75	15	49
Both	24	30	22	25	74	40

**Table 5:** Mean prices per bat for the purchase and sale of fruit bats by active vendors and the mean profit made per bat sold. Prices in US Dollars, per bat carcass. Conversion rate used was 1.41 GHc to 1 USD.

Study site	Number of vendors	Purchase price (USD)	Sale price (USD)	Profit (USD)
Accra	11	0.64	0.86	0.22
Kumasi	4	0.96	1.29	0.33
Nkawkaw	7	0.44	0.65	0.21
Volta	14	0.52	0.72	0.20
Overall	36	0.64	0.88	0.24

## Appendix A Supplementary material

Supplementary data 1 Questionnaires.

## References

[b0005] Bennett E., Blencowe E., Brandon K., Brown D., Burn R., Cowlishaw G., Davis G., Dublin H., Fa J., Milner-Gulland E., Robinson J., Rowcliffe J., Underwood F., Wilkie D. (2007). Hunting for consensus: reconciling bushmeat harvest, conservation and development policy in west and central Africa. Conservation Biology.

[b0010] Brashares J., Arcese P., Sam M., Coppilillo P., Sinclair A., Balmford A. (2004). Bushmeat hunting, wildlife declines, and fish supply in West Africa. Science.

[b0015] Brooke A.P., Tschapka M. (2002). Threats from overhunting to the flying fox, *Pteropus tonganus*, (Chiroptera: Pteropodidae) on Niue Island, South Pacific Ocean. Biological Conservation.

[b0020] Brown D., Williams A. (2003). The case for bushmeat as a component of development policy: issues and challenges. International Forestry Review.

[b0025] Clark G. (1994). Onions are my Husband.

[b0030] Cowlishaw G., Mendelson S., Rowcliffe J.M. (2005). Evidence for post-depletion sustainability in a mature bushmeat market. Journal of Applied Ecology.

[b0035] Cowlishaw G., Mendelson S., Rowcliffe J.M. (2005). Structure and operation of a bushmeat commodity chain in southwestern Ghana. Conservation Biology.

[b0040] Craig P., Trail P., Morrell T.E. (1994). The decline of fruit bats in American Samoa due to hurricanes and overhunting. Biological Conservation.

[b0045] Daszak P., Cunningham A., Hyatt A. (2000). Emerging infectious diseases of wildlife – threats to biodiversity and human health. Science.

[b0050] DeFrees S.L., Wilson D.E. (1988). *Eidolon helvum*. Mammalian Species.

[b0055] Dei G. (1989). Hunting and gathering in a Ghanaian rain forest community. Ecology of Food and Nutrition.

[b0060] Ehrlich P., Wilson E. (1991). Biodiversity studies: science and policy. Science.

[b0065] Epstein J.H., Olival K., Pulliam J., Smith C., Field H., Daszak P. (2009). *Pteropus vampyrus*, a hunted migratory species with a multinational home-range and a need for regional management. Journal of Applied Ecology.

[b0070] Fa J.E., Currie J., Meeuwig J. (2003). Bushmeat and food security in the Congo Basin: linkages between wildlife and people’s future. Environmental Conservation.

[b0075] Fayenuwo J.O., Halstead L.B. (1974). Breeding cycle of straw-colored fruit bat, *Eidolon helvum*, at Ile-Ife, Nigeria. Journal of Mammalogy.

[b0080] Funmilayo O. (1978). Fruit bats for meat: are too many taken?. Oryx.

[b0085] Ghana Statistical Service, 2002. 2000 Population and Housing Census of Ghana: Demographic, Economic and Housing Characteristics. GSS, Accra.

[b0090] Happold D.C.D., Happold M. (1978). The fruitbats of Western Nigeria. Nigerian Field.

[b0095] Harrison M.E., Cheyne S.M., Darma F., Ribowo D.A., Limin S.H., Struebig M.J. (2011). Hunting of flying fozes and perception of disease risk in Indonesian Borneo. Biological Conservation.

[b0105] Hayman, D.T.S., 2008. Lagos Bat Virus Dynamics in Naturally Infected *Eidolon helvum* Fruit Bats in Ghana. First Year Report for University of Cambridge, UK.

[b0110] Hayman D.T.S., Suu-Ire R., Breed A.C. (2008). Evidence of Henipavirus infection in West African Fruit bats. PLoS One.

[b0115] Hayman D.T.S., Fooks A.R., Horton D., Suu-Ire R., Breed A.C., Cunningham A.A., Wood J.L.N. (2008). Antibodies against Lagos bat virus in megachiroptera from West Africa. Emerging Infectious Diseases.

[b0100] Hayman D.T.S., Emmerich P., Yu M., Wang L., Suu-Ire R., Fooks A.R., Cunningham A., Wood J. (2010). Long-term survival of an urban fruit bat seropositive for Ebola and Lagos bat viruses. PLoS One.

[b0120] IUCN, 2010. IUCN Red List of Threatened Species. Version 2010.4. <www.iucnredlist.org> (19.03.11).

[b0125] Jenkins R.K.B., Racey P.A. (2008). Bats as bushmeat in Madagascar. Madagascar Conservation and Development.

[b0130] Jones K., Purvis A., Gittleman J. (2003). Biological correlates of extinction risks in bats. The American Naturalist.

[b0220] Juste J., Fa J.E., Perezdel V.J., Castroviejo J. (1995). Market dynamics of bushmeat species in Equatorial Guinea. Journal of Applied Ecology.

[b0135] Kankam B.O., Oduro W. (2009). Frugivores and fruit removal of Antiaris toxicaria (Moraceae) at Bia Biosphere Reserve. Ghana Journal of Tropical Ecology.

[b0140] Kuzmin I.V., Niezgoda M., Franka R., Agwanda B., Markotter W., Beagley J.C., Urazova O.Y., Breiman R.F., Rupprecht C.E. (2008). Lagos bat virus in Kenya. Journal of Clinical Microbiology.

[b0145] Mbete R.A., Banga-mboko H., Racey P., Mfoukou I., Doucet J., Hornick J., Leroy P. (2011). Household bushmeat consumption in Brazzaville, the Republic of the Congo. Tropical Conservation Science.

[b0150] McConkey K., Drake D. (2006). Flying Foxes cease to function as seed dispersers long before they become rare. Ecology.

[b0155] Mendelson S., Cowlishaw G., Rowcliffe J.M. (2003). Anatomny of a Bushmeat Commodity Chain in Takoradi, Ghana. Journal of Peasant Studies.

[b0160] Mickleburgh S., Waylen K., Racey P. (2009). Bats as bushmeat: a global reivew. Oryx.

[b0165] Milner-Gulland E.J., Rowcliffe J.M. (2007). Conservation and Sustainable Use: a Handbook of Techniques.

[b0170] Milner-Gulland E.J., Bennett E.L., Group S.A.M.W.M. (2003). Wild meat: the bigger picture. Trends in Ecology & Evolution.

[b0175] Mutere F. (1965). Delayed implantation in an equatorial fruit bat. Nature.

[b0180] National Tripartite Committee, 2010. New National Daily Minimum wage is 3.11 Cedis, ed. G.N. Agency.

[b0185] Ntiamoa-Baidu, Y., 1998. Wildlife Development Plan 1998–2003. Sustainable Use of Bushmeat: Wildlife Department, Ministry of Lands and Forestry, Republic of Ghana, Accra 6.

[b0190] Richter H.V., Cumming G.S. (2005). Food availability and annual migration of the straw-colored fruit bat (*Eidolon helvum*). Journal of Zoology.

[b0225] Rowcliffe J.M., Cowlishaw G., Long J. (2003). A model of human hunting impacts in multi-prey communities. Journal of Applied Ecology.

[b0195] Struebig M.J., Harrison M.E., Cheyne S.M., Limin S.H. (2007). Intensive hunting of large flying foxes *Pteropus vampyrus* natunae in Central Kalimantan, Indonesian Borneo. Oryx.

[b0200] Taylor, D.A.R., Kankam, B.O., Wagner, M.R., 2000. The role of fruit bat, Eidolon helvum, in seed dispersal, survival, and germination in *Milicia excelsa*, a threatened West African hardwood. In: Cobbinah, J.R., Wagner, M.R. (Eds.), Research Advances in Restoration of Iroko as a Commercial Species in West Africa, 29–39. Forestry Research Institute of Ghana (FORIG), Kumasi, Ghana, xii+134 pp.

[b0205] Wilcox A.S., Nambu D.M. (2007). Wildlife hunting practices and bushmeat dynamics of the Banyangi and Mbo people of Southwestern Cameroon. Biological Conservation.

[b0210] Wilkie D., Bennett E., Peres C., Cunningham A.A. (2011). The empty forest revisited. Annals of the New York Academy of Sciences.

[b0215] Wolfe N.D., Daszak P., Kilpatrick A.M., Burke D.S. (2005). Bushmeat hunting, deforestation, and prediction of zoonoses emergence. Emerging Infectious Diseases.

