# Supplementary material for: Uncovering the fruit bat bushmeat commodity chain and the true extent of fruit bat hunting in Ghana, West Africa
Source: Biol Conserv. 2011 Dec;144(12):3000–8. doi: 10.1016/j.biocon.2011.09.003 (PMC3323830; doi:10.1016/j.biocon.2011.09.003)
Supplement: Supplementary data 1 — Questionnaires. [file mmc1.pdf]

1. **Do you hunt bats?** [yes, no]
   a. How often do you hunt bats? [daily, 2-5 times a week, weekly, monthly, less often]
   b. How many bats do you catch each time you hunt? [1-2,2-4,5-10, 10-20, more than 20]
   c. (If hunting right now) How many bats did you catch today? [number]
   d. How many bats did you catch last time you hunted them? [number]
   e. Where do you hunt your bats? [location]
   f. What method or methods do you use to catch/kill a bat? [catapulting, gun, snare, poison, scavenge, multiple, other]
2. **Do you sell them or keep them?**
   g. Do you keep any of your captured bats for yourself? [yes, no]
      a. If so, how many did you keep the last time you kept some bats? [number]
   h. How often do you sell bats? [daily, 2-5 times a week, weekly, monthly, less often]
   i. How much of your income do you get from hunting bats? [very little, some, half, most, all]
   j. About how much is that proportion?
   k. How does the time of year affect your income from bats? [open]
   l. How much did you sell your bats for last time you sold them? [GHC]
   m. Has the price of bats gone up, down or stayed the same?
   n. Is the because there are more/fewer bats or because more/fewer people want them?
   o. How far do you have to travel between catching a bat and selling it? [km]
      a. how is the meat transported/care for? [open]
   p. To whom do you sell your bats? [see if I can meet them]
   q. Do you hunt other animals as well? [yes/no] How about "grasscutter" "duiker"
   r. Do you do anything else for money?
3. **Do you sell bats?** [yes/no]
   a. How often do you sell bats? [daily, 2-5 times a week, weekly, monthly, less often]
   b. How much do you sell a bat for? [GHC]
   c. How much did it cost for you to buy the last bats you got? [GHC]
   d. How many bats did you get today? [number]
      i. Yesterday? [number]
      ii. On average? [number]
   e. How many bats did you sell today? [number]
      i. Yesterday? [number]
      ii. On average? [number]
   f. How much of your income do you get from selling bats? [very little, some, half, most, all]
   g. How do you get your bat meat? [killed but unprepared, freshly prepared, smoked, other]
   h. Where do your bats come from (geographically)? [location]
   i. How does the time of year affect your income from bats? [open]
   j. Has the price of bats gone up, down or stayed the same?
   k. Is the because there are more/fewer bats or because more/fewer people want them?
   l. To whom do you sell your bats?
   m. From whom do you get your bats?
4. **Do you eat bats?** [yes/no]
   a. How often do you eat bat meat? [daily, 2-5 times a week, weekly, monthly, less often]
   b. Why do you eat bat meat? [open, but e.g. because its available, cheap, tasty, medicinal]
   c. How is the bat meat prepared [fresh, already butchered, smoked, or fully prepared]?
   d. How much do you pay for a smoked bat?

e. How much do you pay for bat soup?
f. How does the time of year affect your consumption of bats? [open]

5. Do you prepare/butcher bats? [yes/no]

a. How often do you prepare bats yourself (i.e. butcher or smoke them)? [rarely, sometimes, often, always]
b. What do you do? [open, eg remove guts, skin, chop up, smoke]

6 a. Do you think just hunting bats might make you sick? [yes/no]
b. Do you know anyone who has gotten sick from hunting bats? [yes/no]
c. How likely do you think it is that someone would get sick? [very unlikely, unlikely, somewhat likely, likely, very likely]
d. Do you think these activities pose a [a small threat, a significant threat, a serious threat]?

7 a. Do you think that just butchering or preparing bats can make someone sick? [yes/no]
b. Do you know anyone who has gotten sick from butchering or preparing bats? [yes/no]
c. How likely do you think it is that someone would get sick? [very unlikely, unlikely, somewhat likely, likely, very likely]
d. Do you think these activities pose a [a small threat, a significant threat, a serious threat]

8 a. Do you think that just eating bats can make someone sick? [yes/no]
b. Do you know anyone who has gotten sick from eating bats? [yes/no]
c. How likely do you think it is that someone would get sick? [very unlikely, unlikely, somewhat likely, likely, very likely]
d. Do you think these activities pose a small threat, a significant threat, or a serious threat?

9. Do you feel that people want bat meat more than, less than or the same as they did five years ago?

10. Who do you think most commonly eats bats?

11. Which do you prefer, bush meat or domestic meat?

12. Do you ever trade bats for something other than money, or use something other than money to trade for a bat? [yes/no]
i. What did you trade?

13. Can you put these cards in order of which you like to eat best to which you like to eat least? [grasscutter, bat, pork, chicken, beef, goat, duiker]

14. Can you put these cards in order of which you eat most often to which you eat least often? [grasscutter, bat, pork, chicken, beef, goat, duiker]

Demographic:
Sex
Age
Education
Group
From
Lived
